# Antibiotic Tolerance of *Staphylococcus aureus* Biofilm in Periprosthetic Joint Infections and Antibiofilm Strategies

**DOI:** 10.3390/antibiotics9090547

**Published:** 2020-08-27

**Authors:** Fabien Lamret, Marius Colin, Céline Mongaret, Sophie C. Gangloff, Fany Reffuveille

**Affiliations:** 1 EA 4691 Biomatériaux et Inflammation en Site Osseux (BIOS), Université de Reims Champagne-Ardenne, SFR Cap Santé (FED 4231), 51097 Reims, France; fabien.lamret@univ-reims.fr (F.L.); marius.colin@univ-reims.fr (M.C.); celine.mongaret@univ-reims.fr (C.M.); sophie.gangloff@univ-reims.fr (S.C.G.); 2 Service Pharmacie, CHU Reims, 51097 Reims, France

**Keywords:** *Staphylococcus aureus*, biofilms, antibiotic, resistance, tolerance, periprosthetic joint infections

## Abstract

The need for bone and joint prostheses is currently growing due to population aging, leading to an increase in prosthetic joint infection cases. Biofilms represent an adaptive and quite common bacterial response to several stress factors which confer an important protection to bacteria. Biofilm formation starts with bacterial adhesion on a surface, such as an orthopedic prosthesis, further reinforced by matrix synthesis. The biofilm formation and structure depend on the immediate environment of the bacteria. In the case of infection, the periprosthetic joint environment represents a particular interface between bacteria, host cells, and the implant, favoring biofilm initiation and maturation. Treating such an infection represents a huge challenge because of the biofilm-specific high tolerance to antibiotics and its ability to evade the immune system. It is crucial to understand these mechanisms in order to find new and adapted strategies to prevent and eradicate implant-associated infections. Therefore, adapted models mimicking the infectious site are of utmost importance to recreate a relevant environment in order to test potential antibiofilm molecules. In periprosthetic joint infections, *Staphylococcus aureus* is mainly involved because of its high adaptation to the human physiology. The current review deals with the mechanisms involved in the antibiotic resistance and tolerance of *Staphylococcus aureus* in the particular periprosthetic joint infection context, and exposes different strategies to manage these infections.

## 1. Role of Bacterial Biofilm in Prosthetic Joint Infections

### 1.1. Definition of Biofilm

An accurate definition of the biofilm is still debated in the scientific community. Bill Costerton used the term “biofilm” for the first time in 1978 and stated a definition in 2002: “biofilm is a microbially derived sessile community characterized by cells that are irreversibly attached to a substratum or interface or to each other, embedded in a matrix of extracellular polymeric substances that they have produced, and exhibit an altered phenotype with respect to growth rate and gene transcription” [1]. Hans Curt Flemming recently completed this definition by describing biofilms as “an emergent form of bacterial life, in which communal life is completely different from bacteria that live as free-living cells” [2].

**Figure 1:**
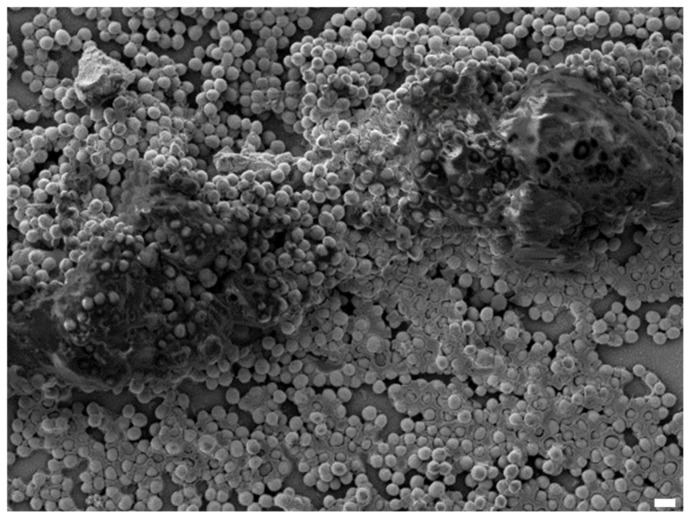
Scanning electron microscopy acquisition of *S. aureus* biofilm in vitro. Scale bar = 1 μm.

Even if the biofilm definition evolves, the biofilm life cycle has been well known for many years. It starts with the adhesion of bacteria to a surface in response to external stimuli or stress [3]. In the case of the *Staphylococcus aureus* (*S. aureus*) adhesion to biomaterials (Figure 1), this attachment can be passive or active. Passive attachment is characterized by a reversible adhesion mechanism involving van der Wall, acid–base, and electronic interactions, while active adhesion involves an array of microbial surface components recognizing adhesive matrix molecules (MSCRAMMs) to engage in irreversible attachment [4,5]. Thus, bacteria can colonize various environments. Unfortunately, biofilms are omnipresent in hospitals, where many surfaces allow biofilm formation. Foreign medical devices are especially involved, such as catheters, pacemakers, or bone and joint prostheses, which are regularly colonized by bacteria [6]. When bacteria are finally irreversibly anchored to a surface, the maturation of biofilm can occur through the bacterial production of a matrix, which is mainly composed of water, exopolysaccharides, proteins, and extracellular DNA. A matrix allows bacteria to proliferate in an organized and structured community [3]. Moreover, the matrix protects embedded bacteria from immune cell attacks, mediates further bacterial adhesion, provides mechanical stability, and retains essential nutrients and enzymes [7]. When the biofilm is mature enough, the release and dispersion of bacteria entrapped in the biofilm structure can eventually occur under specific conditions, such as a decrease in stress factors. Newly free bacteria are thereby able to colonize new surfaces to find new nutrients in, for example, the hematogenous way [3]. The bacterial communication mode called quorum sensing is particularly important to achieve this step. The most known quorum-sensing system in *S. aureus* is the Agr (accessory gene regulator) program. Its regulation is based on cell density according to the state of the biofilm, and can control the secretion of cell surface proteins, surface adhesins, or virulence factors, and thereby is involved in both biofilm maturation and dispersion through bacterial egress [3].

An important characteristic of biofilms is their heterogeneity, which is both spatial and cellular. This heterogeneity is due to numerous factors that interact at different concentrations through the biofilm thickness. Thus, gradients of nutrients, oxygen, or antimicrobial molecules (among other factors) are present, and they affect bacteria according to their position in the biofilm [8]. Matrix and bacteria are intertwined to organize a mature biofilm and, in this state, bacteria can metabolically be very different from one another. Even in monospecies biofilms, bacteria in various metabolic states co-exist and are spatially organized in the way mentioned before [9]. This results in four main metabolic states that can be found within the biofilm: aerobic, fermentative, dormant, or dead [10]. These various states can lead to several particular bacterial phenotypes, such as persister cells, small colony variants (SCVs), or viable but non-culturable (VBNC) bacteria [11,12,13,14]. Persister cells are bacteria already present in the planktonic population and can withstand high concentrations of antimicrobial drugs. These cells are found in an increased frequency in biofilms [15]. SCV bacteria will be addressed further later for their implication for biofilm formation and antibiotic escape. The VBNC state corresponds to bacteria which are less metabolically active and fail to grow on routine bacteriological media; this state can be induced in biofilms by antibiotic exposure [16,17]. As a large panel of antibiotics rely on active bacterial cells, these metabolic adaptations are a key factor in bacterial tolerance to antibiotics [18].

### 1.2. Biofilm and Clinical Impact

An important part of bacterial infections, estimated to be 80%, involves biofilm formation, which is not surprising given the numerous advantages that this lifestyle offers to pathogens [3]. Indeed, biofilm firstly acts as a shield against the host immune system, allowing bacteria to elude innate and adaptive host defenses, at least partially [3,4]. Overall, microorganisms use a large set of strategies to thwart immune defenses, such as internalization in host cells, toxin production, and immune response hijacking. Another strategy inherent to biofilm is the emergence of SCVs. These bacteria are phenotypically characterized by a smaller colony formation, resulting from bacterial slow metabolism—i.e., slow growth and slow cytotoxic factor secretion [4,19]. The phenotype switch to SCV and the bacterial internalization in host cells are two strategies that increase the survival time of bacteria within the host [4]. Moreover, each step of biofilm development is problematic, as an immature biofilm is less detectable by the immune system and a mature one is more protected from immune attacks.

Biofilms hide a complex and organized structure, responsible for antibiotic resistance and tolerance (concepts detailed in Section 2.1 and Section 2.2), in addition to immune system evasion. One of the worst consequences of biofilm infections is that they lead to an overuse of antibiotics without, in most cases, any therapeutic effect, which possibly enhances the emergence of antibiotic resistance.

First, the proximity between bacteria in the biofilm state induces the possibility of antibiotic resistance gene exchange [20]. Then, the biofilm state allows bacteria to be tolerant to antibiotic treatment due to the structural and metabolic heterogeneity of the biofilm [21]. The concept of resistance will be detailed in Section 2 of this review.

Staphylococcal species are particularly involved in various biofilm-related infections. Infections of skin and soft tissues, endocarditis, urinary tract infections, and cystic fibrosis are some examples, but staphylococcal species, and especially *S. aureus*, are well known to be the major cause of bone-related infections, such as osteomyelitis and implant-related infections. The biofilm characteristics explain the infection rate in the case of implant-related bone infections, as implants seem to facilitate biofilm formation [22].

### 1.3. Periprosthetic Joint Infections and Biofilm

#### 1.3.1. Periprosthetic Joint Infections: Definition and Clinical Impact

Biofilms are notably involved in osteomyelitis, an infection of the bone characterized by bacterial colonization leading to inflammatory reaction and the resorption of bone [23]. The bacterial presence can be due to an adjacent contaminated bone, a bacteremia, or a colonized implant [24]. The latter, named Prosthetic Joint Infections or Periprosthetic Joint Infections (PJIs), is defined as an osteomyelitis infection characterized by a post-operative infection that occurs after the prosthetic joint surgery [24]. The need for bone and joint prostheses is currently growing due to population aging, and it will probably continue to increase in the future [25,26].

Even taking into account prophylaxis and aseptic surgical technique, the percentage of PJIs following intervention do not decrease under 1–2%, depending on the bone or joint location [27,28]. In the case of infection, the presence of a prosthesis allows bacteria to adhere to it, develop a biofilm, and trigger an infection of the surrounding tissues. PJIs then lead to prosthesis loosening, amputation, or even the death of patients in the worst scenario, and represent high costs for society [26,29].

*S. aureus*, including methicillin-sensitive (MSSA) and methicillin-resistant (MRSA) strains, is the most common species responsible for this kind of infection, and both MSSA and MRSA can be healthcare-related or community-acquired [30]. Coagulase-negative *Staphylococcus*, *Streptococcus*, and *Enterococcus* species and *Cutibacterium acnes* are other species often involved in PJIs, but MRSA alone is responsible for around 50% of all PJIs [27,31,32].

Bone infections due to implanted materials (prosthesis, fixative, material, or bone substitutes) can be acute or chronic. The definition of these two states is not universal, and depends on the considered bone and joint location [33]. Acute implant infections are mainly related to peri-operative or contiguous contamination from adjacent tissues, whereas chronic infections are mostly related to a hematogenous spread from a distant infectious site [26]. Chronic bone infections are due to unsuccessful treatment and are characterized by the persistence of bacteria, areas of dead bone, so-called sequestra, periosteal new bone formation, fistula, and low-grade inflammation [23]. Both acute and chronic infections are linked to biofilm presence, but there is no precise data on the actual number of infections especially due to the presence of a biofilm [23,34].

Reinfections occur very frequently after revision surgery, with 33% of revisions leading to relapse, and are quite significant and depend on the surgical procedure used [23,27]. This high rate of relapses may be explained by the presence of bacterial biofilm found around the infectious site, which elucidates bacterial persistence and the recurrence of reinfection. Masters et al. highlighted three main reservoirs of biofilm: staphylococcal abscess communities in the local soft tissue and bone marrow, glycocalyx formation on the implant hardware and necrotic tissue, and the colonization of the osteocyte lacuno-canalicular network (OLCN) of cortical bone [27].

In addition to being able to form biofilms, bacteria can also colonize human cells. In vitro and in vivo studies showed that *S. aureus* can persist intracellularly in a lot of cells found in the bone environment, including bone cells such as osteoblasts, osteoclasts, and osteocytes, and also in immune cells such as Kupffer cells and macrophages in the case of systemic infection [35,36,37,38,39]. However, continued research is warranted to highlight the impact of internalization in each cell type on the durability and the severity of the infection. Indeed, it is very difficult to observe this phenomenon in histological sections of affected patients. Thus, this phenomenon of internalization in patients’ bone cells is currently being debated.

Another important parameter during PJIs is the immune system reaction. Indeed, after surgery and during infection establishment, the immune system reacts not only to bacterial presence but also to the implant surface. This phenomenon is called foreign body response and leads to an inflammatory response involving the coagulation cascade, complement system, platelets, and immune cells, particularly neutrophils [4,29,40]. This inflammation, which occurs during surgery, can normally lead to physiological bone healing, but can also induce bone resorption accentuated during bone infections [41]. Moreover, the bacterial-killing capacities of neutrophils are reduced by material activation [4].

#### 1.3.2. Patients’ Risk Factors

From a medical context, patients can present risk co-factors, increasing PIJ occurrence, as highlighted in clinical studies. To name a few, diabetes, obesity, malnutrition, smoking, and low vitamin D levels are modifiable risk factors involved in infection onset [42,43,44,45,46,47]. Nair et al. also described risk factors among older patients, summarizing the modifiable and non-modifiable risk factors [48]. To know and to manage these risk factors can help to prevent the occurrence of some infections. The link between these factors and biofilm development remains unknown.

#### 1.3.3. Clinical Signs

The knowledge of PJIs allows a specific focus on different symptoms after surgery. Attention will be paid to pain, warming, swelling, and fever, which are alarming symptoms revealing infection by highly virulent bacteria such as *S. aureus* [23]. However, diagnosis can be challenging when low-grade clinical symptoms are observed or when symptoms appear a long time after surgery, such as for chronic infection, which is characterized by the silent presence of bacteria [49].

#### 1.3.4. Current Strategies vs. Biofilm PJIs

Once the diagnosis has been made and the PJI turned out, debridement, antibiotics, irrigation, and retention (DAIR) are generally the treatments given to patients depending on the infection severity—i.e., the stability of the implant, the pathogens involved, and their susceptibility to antibiotic treatments and the onset of infection [23,27,28,44]. One- or two-stage arthroplasty exchange, arthroplasty resection without reimplantation, or even amputation are performed if needed—for instance, if the causative pathogen is an MRSA or in the case of mature biofilm formation on the implant [23,26]. Treatment failures because of reinfection depend on the surgical procedure and joint localization too, and they are estimated to reach 8% for the hip and knee [24]. Algorithms for the selection of the medical/surgical treatment strategy and suggestions for appropriate antibiotic treatments are used as a guide in the selection of the most suitable procedure [50].

Owing to therapeutic failures, it seems important to understand why molecules such as antibiotics are inefficient at eradicating biofilm infections through a better understanding of biofilm formation and the protection mechanisms involved, especially in PJIs.

## 2. Antibiotic Failure in PJI Due to *S. aureus* and Biofilm Role

In this review, we focus on *S. aureus* because this Gram-positive aero-anaerotolerant coccus is one of the most prevalent bacteria related to healthcare-associated infections, especially PJIs. This is probably due to its high potential for metastatic infection, high affinity for foreign material, and extraordinary ability to develop antibiotic resistance [18,51]. The prevalence of *S. aureus* can also be explain by the skin and soft tissue colonization leading to infection through wounds and the hematogenous way [52,53,54]. Thus, *S. aureus* is the most common species causing PJIs, as recent reviews estimate its incrimination between 27% to 43.6% [26,31]. However, the epidemiology of bacteria involved in PJIs depends on the joint concerned and even the country [31,55,56].

In addition to asymptomatic carriers in the population, *S. aureus* possesses, develops, or acquires antibiotic resistance genes [18]. MRSA involved in PJIs can reach 50% of cases, complicating the treatment of such infections [27,57].

Studies have shown that MSSA and MRSA phenotypes have different behaviors during infection, including different biofilm production and different pathways involved in biofilm formation or virulence [58,59,60]. As already known, most antibiotics treatments are not effective against *S. aureus* biofilms. Worse, several studies have shown that antibiotics treatments can induce biofilm formation [61,62]. For now, rifampicin (rifampin) is the most efficient antibiotic against *S. aureus* biofilms; presents a good penetration into bone tissues; and shows good efficacy in vitro, in vivo, and in clinical PJIs. However, it should be used with care because of resistance emergence [49,63,64]. In France, oxacillin, cloxacillin, or cefazolin are recommended for the intravenous initial treatment of multiresistant staphylococci, followed by the per os administration of ofloxacine and rifampicin [65]. Unfortunately, this treatment remains ineffective against advanced cases of infection, and surgical management is required.

Thus, in spite of the discovery of many antimicrobial molecules, it is not possible to decrease or even eradicate the resistance of PJI to already used therapeutic treatments.

First, it is important to highlight the difference between antibiotic resistance and tolerance. Brauner et al. proposed the definitions and distinctions of these mechanisms, and highlighted the importance of clearly discriminating between these terms [66]. Resistance describes the inherited ability of microorganisms to grow despite high concentrations of an antibiotic [66]. Tolerance describes the capacity, whether inherited or not, of microorganisms to survive a transient exposure to high antimicrobial concentrations. These definitions are based on minimum inhibitory concentration (MIC) and minimum duration for killing (MDK) determination [66]. In the case of antibiotic resistance or tolerance to bacteria within a biofilm, the definition has to be adapted.

### 2.1. Antibiotic Resistance

According to scientific estimation, 10 million people will die because of antibiotic resistance by 2050 [67]. Antibiotics were revolutionary molecules discovered in the 1940s, specifically targeting bacteria without affecting human cells. However, antibiotics are overused in the medical field and animal farming [67]. The overuse of antibiotics also occurs in the case of chronic infection, whose origin is the installation of a biofilm. It is suggested that the golden age of antibiotics will be over soon and bring a post-antibiotics era [18,68]. To curb the emergence and spread of bacterial resistance, there is an urge to improve antibiotic prescribing practices and find new strategies to replace or enhance antibiotics, for which the discovery of new molecules is low or not adapted to treat the biofilm state [68]. As each infectious environment is specific, it is necessary for therapeutic molecules to be tested through suitable models.

Antibiotic resistance is commonly described as a change in the bacteria that is transmissible to the next generation, and can be intrinsic or acquired [10,69]. Different mechanisms of antibiotics resistance are well-described: (i) enzymatic drug modification and inactivation, (ii) enzymatic modification of the drug binding site, (iii) drug efflux, (iv) bypass mechanisms involving the acquisition of a novel drug-resistant target, (v) the displacement of the drug to protect the target [18]. The acquisition of resistance can operate through mutations of the bacterial genome or by horizontal gene transfer, which is the acquisition of new genes from other bacteria.

The acquisition of resistance by mutation can result from (i) an alteration of the drug target that prevents the inhibitor from binding; (ii) the derepression of chromosomally encoded multidrug resistance efflux pumps; and (iii) multiple stepwise mutations that alter the structure and composition of the cell wall and/or membrane to reduce the drug access to its target [18].

Horizontal genes transfers are the most common mechanism for bacteria to acquire resistance. In biofilm, the high density of bacteria increases this phenomenon, favoring the release and acquisition of genetic material [20,70]. This occurs through DNA fragments sequestrated in the matrix, which can be transferred between bacteria [71]. Therefore, biofilm can even be seen as a library of antibiotic resistance genes [7].

Thus, bacteria in biofilm are more likely to acquire resistance, especially against antibiotics. In addition, in PJI the biofilm generates oxidative stress, which enhances the bacterial mutability and thus the chances of resistance genes acquisition [72]. Moreover, the treatment of PJI with high concentrations of antimicrobial molecules induces a pressure which selects resistant bacteria.

To complete the picture, efflux proteins, which are initially responsible for the extrusion of antibiotics from bacteria, are also linked to biofilm formation. Some of these efflux pumps are overexpressed under the biofilm lifestyle, thereby causing increased antibiotic resistance [73,74,75].

### 2.2. Antibiotic Tolerance

Olsen et al. described that antibiotic resistance is a natural phenomenon, independent from human activity, even if it is now a hot field due to the recent emergence of a great amount of resistant pathogenic bacteria [9]. Antibiotic tolerance, meanwhile, is a transitory and reversible phenotypic state. Biofilm antibiotic tolerance is also a natural state, due to the biofilm mode of growth. Many components of the biofilm and the bacterial behavior itself induce antibiotic tolerance and are described below.

#### 2.2.1. Matrix

First, the matrix produced by bacterial cells has a protection function and serves as a scaffold for biofilm formation. Second, the nutrients and antimicrobials can be sequestered close to bacteria, and this retention of drugs by matrix elements can induce resistance to antibiotics [7,9,76]. Within the *S. aureus* biofilm matrix, extracellular DNA (eDNA) originates from dead bacteria and stabilizes the biofilm structure. The eDNA also inhibits the penetration of antibiotics into the biofilm and acts as a cation chelator [77]. As already described above, it is also an important mechanism for horizontal gene transfer [70].

The main point is that the composition of the matrix slows down the antibiotic penetration, leading to sub-MIC concentrations, which are known to lead to favor biofilm formation; to inducing mutagenesis; and to increasing resistance mechanisms [9,78]. However, even if some antibiotics can reach the deeper layers of biofilm, viable bacteria are still recovered, indicating that the matrix’s partial impermeabilization is not the only mechanism involved in antibiotic tolerance [34].

#### 2.2.2. Metabolism

Within biofilms, bacteria present different growth rates, depending on the sub-population and factor gradients such as oxygen or nutrients, impacting the antibiotic activity. Indeed, due to fewer nutrient being available in biofilm, bacteria have to adapt their metabolism to survive. Metabolic activity hugely influences the antibiotic susceptibility of bacteria, and biofilms shelter heterogeneous sub-populations under different altered metabolisms [10]. Often localized in the inner part of biofilm, less active bacteria are also less sensitive to many antibiotics targeting the growing metabolism [24]. However, slowing down their metabolism within biofilms is not the only way bacteria can escape antibiotics, as specific pathways can also lead to antibiotic evasion.

As biofilm emerges as a stress response to hostile environment, different SOS systems are triggered in the presence of stressful conditions, such as antibiotic treatments, starvation, or oxidative stress, to repair damaged DNA caused by these threats [79]. Starvation and the associated SOS response can mediate biofilm-specific tolerance to antibiotic such as ofloxacine, and it was also shown that subinhibitory concentrations of quinolones can induce an SOS response and elevate the mutation rate [80,81]. Generated by the host, reactive oxygen species are also linked with antibiotic tolerance during systemic *S. aureus* infections [82]. The SOS response also affects the expression of virulence factors, mutagenesis, and the frequency of SCVs [79,83]. This phenotype switch in *S. aureus* is described as a consequence of drug treatments, reactive oxygen species, low pH, and starvation, and is also linked to the internalization ability [12,36].

Another metabolism adaption found in biofilm is the toxin/antitoxin (TA) system activation, which is suspected of leading to persister bacteria formation [84,85]. Bacterial toxin-antitoxin (TA) modules consist of a stable toxin that inhibits bacterial cell growth by disrupting a vital process and a labile anti-toxin that counteracts the toxin and protects the cell [84,85,86]. In *S. aureus*, yoeB is a TA system leading to persister switch [84]. Persister cells are defined as a small subpopulation of bacteria that has entered a slow growing or starving state and that is highly tolerant to killing by antibiotics [9]. Persisters are less sensitive to antibiotics because they are not undergoing cellular activities that antibiotics can corrupt [84]. This phenotype is also found in the planktonic phase before biofilm formation and can repopulate the infectious site or be responsible for relapse after antibiotic treatments, and can thereby be involved in the persistence of biofilm infection [9,11,87]. MazEF is another TA system involved in biofilm formation and biofilm antibiotic tolerance [85]. Notably, by promoting the persister phenotype, TA systems increase antibiotic tolerance.

Finally, the stringent stress response has a key role in biofilm formation and dispersal, but also in antibiotic tolerance [87,88]. The stringent response is mediated by the alarmone guanosine tetraphosphate (ppGpp), and the relA gene is the key player in this alarmone synthesis in *S. aureus* [12,88]. This pathway results from nutrient limitation and allows bacteria to save energy and to conserve cellular resources, determining the resistance level of an MRSA strain [89]. By modulating the ppGpp level, which is related to the susceptibility to cell-wall active antibiotics, the stringent response is involved in antibiotic tolerance [90].

Thus, antibiotic tolerance and resistance are different useful tools found in biofilms and used by bacteria to survive antibiotics treatments. Altogether, these mechanisms explain why antibiotic treatments are not suitable against the majority of PJIs.

### 2.3. The Persistence of PJI

Because of the *S. aureus* ability to persist in the periprosthetic joint environment, the bone microenvironment surrounding the implant is suspected to play a non-negligible role in persistence by promoting bacterial infection or relapse. More precisely, this specific environment is characterized by the implant presence, the immune and bone cells’ proximity, a poor vascular supply, and an inflammatory circumstance [29,31,91]. In this context, antibiotics and immune cells hardly reach the bone and periprosthetic site, leading to sub-MIC concentrations of antibiotics, favoring bacterial resistance acquisition and biofilm formation [9,78]. Furthermore, the implant itself presents an attractive surface, allowing bacterial colonization and generating an inflammatory response from the host, creating a favorable context for biofilm establishment [29].

**Figure 2:**
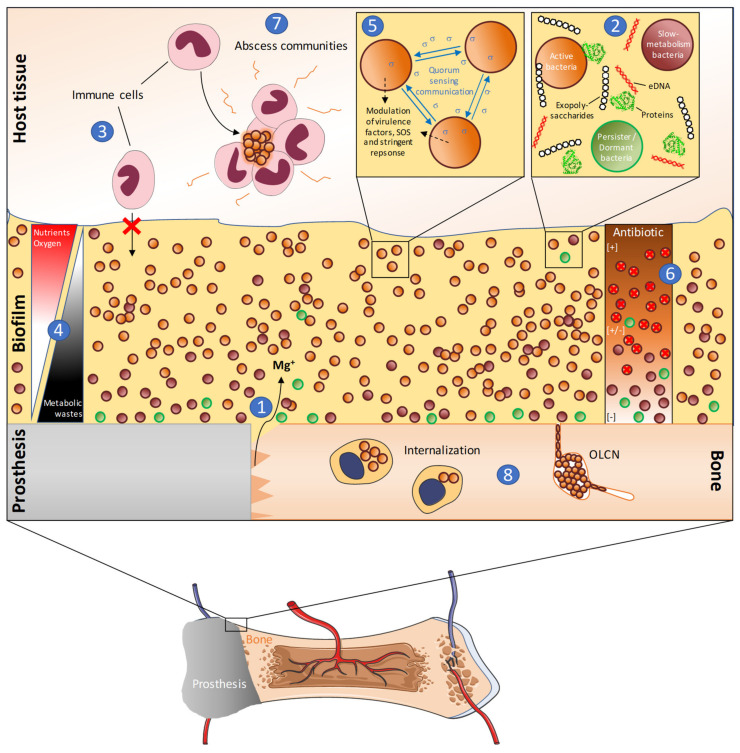
Representation of Periprosthetic Joint Infection involving *S. aureus* biofilm and the mechanisms of persistence. (**1**) Following surgery, traumatized bone releases several factors such as magnesium, inducing a specific microenvironment where *S. aureus* biofilm can develop. (**2**) Biofilm is composed of bacteria under various metabolisms and embedded in a matrix essentially composed of exopolysaccharides, extracellular DNA (eDNA), and proteins. (**3**) Antibacterial action of all types of immune cells is obstructed by the biofilm. (**4**) Biofilms’ semi-permeable structure induces the formation of diffusion gradients of nutrients and oxygen, found in higher concentrations on the upper layers of the biofilm, and an opposite gradient of metabolic wastes that accumulates in the deeper layers. (**5**). Communication signals (quorum sensing) are produced and captured by bacteria. In addition to stress factors, these signals regulate the metabolism activity, the production of virulence factors, and other bacterial responses. It results in metabolic heterogeneities within the biofilm. (**6**) Antibiotics partially penetrate into the biofilm, resulting in a high concentration in the upper parts and progressively lower concentrations in deeper parts. While active bacteria in the upper parts are efficiently killed, the sub-Minimum Inhibitory Concentrations of antibiotics in the inner parts allow bacteria to survive, especially those with slow metabolisms, such as persisters. (**7**) Even when surgery is performed, *S. aureus* can persists under abscess communities in the surrounding tissues of the bone or (**8**) by internalizing within bone cells such as osteoblasts, or even by colonizing the osteocyte lacuno-canalicular network (OLCN). These strategies allow bacteria to escape or hide from the immune system and can act as sources for future reinfections.

Understanding this environment is the key to fighting PJIs. For this purpose, there is an emergent need to reliably characterize the parameters influencing biofilm formation, and to design adapted models relevant to the clinical reality. Such models could be mandatory to analyze the antimicrobial efficiency and improve other antibiofilm strategies. To help to understand PJIs’ persistence and recurrence, Figure 2 represents a non-exhaustive overview of several factors that play a role in the tolerance and the persistence of PJIs [10,27,92,93].

## 3. Emergent Antibiofilm Strategies in Periprosthetic Joint Infection

To perform a clinical diagnosis and select an adapted treatment, the determination of minimal inhibitory concentration is usually performed on planktonic form, but this method is completely unsuitable regarding biofilm-related infections with high protections against antibiotics [34]. Tasse et al. have already tested a new way to assess biofilm resistance toward antibiotics (new Antibiofilmogram^®^ tests) and published a study on biofilm MIC, called bMIC, which corresponds to the concentration of antibiotics that prevents biofilm establishment [94,95]. Nevertheless, definitive evidences of correlation between in vitro robust biofilm of a specific strain and its ability to cause PJI are lacking, as most of the models showed important discrepancies with the clinical reality. Research has to go further by finding specific antibiofilm strategies, adapted to the PJI site.

**Figure 3:**
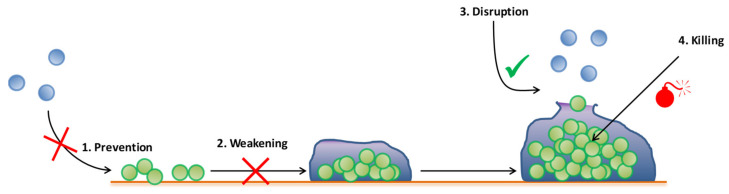
Summary of different ways to fight biofilm during its lifecycle.

Several strategies exist in order to fight bacterial infections, and more particularly bone biofilm-related infections [3]. Each of these strategies focuses on a specific stage of the biofilm lifecycle. Briefly, we resume four ways to fight biofilm in Figure 3. “Prevention” consists of inhibiting bacterial adhesion and at least biofilm formation. Thus, bugs are maintained under their planktonic form in order to maintain their susceptibility to antibiotics and to the immune system. The “weakening” strategy corresponds to avoid the biofilm maturation. “Disruption” is the fact of promoting biofilm dispersion on advanced stage biofilms. Finally, “killing” corresponds the eradication of a preformed biofilm, but remains the most challenging strategy, as mature biofilms are currently untreatable. Additionally, these two last strategies can eventually lead to the release of a large amount of bacteria from the biofilm, and can thereby represent a high risk for patients—for example, by inducing a septic shock. To fight an already formed biofilm, the better scenario is to kill embedded bacteria hidden within the biofilm while reducing dispersion [96].

### 3.1. Antibiofilm Molecules

#### 3.1.1. Antibiotics (Combination, Anti-MRSA)

To circumvent the problem of antibiotic resistance and tolerance of biofilms, different strategies are proposed. Since the late 1980s, the discovery of new antibiotics has drastically decreased [18]. However, new antibiotic development still continues with the will to find new targets and thwart antibiotic resistance and tolerance [18]. For instance, scientists are developing new molecules targeting less common metabolite mechanisms, such as fatty acid and teichoic acid biosynthesis, and dormant bacteria such as persisters [18]. It is also important to note that, as for free bacteria, the concentration of antibiotics and the time of antibiotic application are crucial parameters to take into account in the fight against biofilm structures [97].

The combination of antimicrobial and antibiofilm molecules appears as a good strategy to disrupt the biofilm. It offers better effectiveness of antimicrobial agents, and a lower opportunity for antibiotic resistance acquisition to occur within biofilm [68]. To date, rifampicin, doxycycline, and daptomycin seem to be the most effective antibiotics against *S. aureus* clinical isolates found in biofilm-related PJIs [98]. Enhancing antibiotic penetration or disturbing the stringent response, which both are involved in antibiotic tolerance due to the biofilm state, seem to be good strategies to improve antibiotic efficiency [99]. Effective combinations of antibiotics need to inhibit biofilm formation and more precisely adhesion, while demonstrating a good bactericidal activity in biofilm [34].

In the case of PJIs, optimal treatments require antibiotic combinations, especially to target slow- or not-growing bacteria [34]. Against *S. aureus* biofilm, rifampicin is the most common partner drug of effective combinations which were demonstrated in vitro and in vivo [34,100]. New antibiotics, such as oritavancin, have to deal with MRSA and vancomycin-resistant *S. aureus*, and also with intracellular bacteria—i.e., internalized bacteria in host cells—as rifampicin does, in order to show a better bacterial burden clearance [64,101,102]. To enhance these results, new antibiofilm molecules should probably be tested in combination with a classic antibiotic such as rifampicin in order to look for synergistic effects and act against all types of metabolism activities. Furthermore, the acquisition of antibiotic resistance can lead to increased susceptibility to other antibiotics. This phenomenon is named collateral sensitivity and can be used to deal with antibiotic resistance by combination or sequential antibiotic therapy [103].

#### 3.1.2. Antimicrobial Peptides (AMPs)

Antimicrobial peptides (AMPs), also named host defense peptides (HDPs), are natural or synthetic molecules that are promising to fight bacteria and arise as a good alternative to antibiotics [88]. Briefly, once linked to their target, AMPs display membrane-disturbing properties, but also intracellular activities due to their capacity to freely translocate across the bilayer membrane [88]. AMPs differ from conventional antibiotics, as they target a less specific mechanism than antibiotics, reducing selective pressure and the risk of resistance mechanisms’ emergence [88,104].

AMPs are also promising because their activity as antibiofilm molecules can be independent of their activity against planktonic bacteria [88]. Associated with their low cytotoxicity and their activity against MRSA biofilm in vitro and in vivo, these characteristics arise in AMPs as serious candidates to investigate [101,105]. They also showed efficiency against slow-growing or dormant bacteria [106]. Many different AMPs were discovered and exhibit immunomodulatory and anti-biofilm activities [88]. They can potentially be used in combination with antibiotics to create synergistic effects to fight biofilm, due to the great number of available molecules to date and the different target sites and mechanisms of action [88,96]. Coating AMPs on bone and joint prosthesis could be a good strategy to prevent the adhesion of bacteria and to weaken the biofilm structure.

### 3.2. Immune Modulation and Immunotherapy

As mentioned earlier, bone surgery induces a specific immune context. Modulating the immune system and the inflammation in order to promote natural human body defenses could be a helpful way to manage biofilm-related infections. In this context, molecules such as AMPs can turn out to be great candidates, as they present immunomodulatory properties [88]. Vaccines with monoclonal antibodies targeting specific *S. aureus* proteins, such as surface protein A, are also an option [101]. Such a strategy can be again used in combination with antibiotic treatments [101]. Seebach et al. showed some key points to investigate the redirection of the immune system during implant-related bone infection [23]. Indeed, there is a communication between cells surrounding the periprosthetic environment in case of PJIs, i.e., *S. aureus* bacteria, immune cells, and bone cells [23,29]. Characterizing these interactions could help to understand and counteract the persistence of *S. aureus* in the periprosthetic environment.

### 3.3. Phage Therapy

Bacteriophages are viruses specifically targeting bacterial cells. Due to this specific property, the idea of using bacteriophages to address bacterial infection issues is currently growing as an ambitious alternative strategy. Associated with antibiotics treatments, phage therapies are promising to thwart antibiotic tolerance and resistance, as these two weapons against biofilm are based on entirely different mechanisms of action [97,107]. Furthermore, association with antibiotics have the benefit to improve the effectiveness of these molecules without using high doses, which could help to lower the resistance mechanisms and decrease toxic side effects [107]. It is important to note that the time and order of treatment are important to improve antibiofilm properties of combination with antibiotics [107,108]. As host cells can potentially represent a reservoir of internalized bacteria and a source of reinfection, targeting internalized bacteria in cells would be a huge advantage, but there is a lack of efficiency of bacteriophages against internalized bacteria such as *S. aureus* [109].

### 3.4. Prosthesis Management by Coating Surfaces

Prosthesis often represent ideal surfaces for bacterial adhesion, as implants are often composed of cobalt-chrome, titanium, ceramic, and polyethylene and can be colonized by bacteria [110]. However, strategies exist to turn inert surface into biomaterial surface, through management or antimicrobial molecules coating which allow the production of functionalized prosthesis able to prevent bacterial adhesion. “Biomaterials” journal defines biomaterial as “a substance that has been engineered to take form which, alone or as a part of a complex system, is used to direct, by control of interactions with components of living systems, the course of any therapeutic or diagnostic procedure” [111]. Surface area, roughness, energy and hydrophilicity are important parameters to manage protein adsorption and microbial attachment [112].

Different strategies are investigated in order to limit bacterial adhesion on bone and joint prosthesis using antimicrobial molecules coating. Indeed, it is possible to cover prosthesis surface with antimicrobial molecules that will be bound or released. However, such technology can induce antibiotic resistance and antibiotic-loaded scaffolds used carriers such as chitosan, hydroxyapatite or collagen which may induce tissue toxicity [113].

Adaptive properties are also investigated, such as stimulus-responsive biomaterials [5]. These biomaterials release antimicrobials once bacteria interact with them, in order to inhibit bacterial adherence and biofilm formation [5]. Antimicrobial agents can be RNA, antibiotics, AMPs and nanoparticles. However, reduced bacterial adhesion can also reduce the tissue integration, highlighting the complexity to both manage infection and enhance prosthesis integration [5]. In consequence, the “race to the surface” between host cells and pathogens is a crucial phenomenon to include in research. Indeed, to address a preformed biofilm or a mature biofilm is complicated and the best way to fight biofilm-related infections seems to be to inhibit early stages of biofilm formation [34]. A possible way to aim this goal is to promote host cell adhesion. In the context of PJIs, host cells and bacteria co-exist, and both populations are in competition for the colonization of the prosthesis surface. This is why co-cultured systems are performed, in order to better characterize this “race to the surface” [114]. If host cells first colonize the implant, such as bone cells in the case of PJIs, bacterial colonization would be reduced. The colonization of an implant by host cells is an important point to evaluate the efficiency of biomaterials. To increase the biocompatibility and tissue integration, pro-osteoblast molecules can be added to create a bifunctional surface [114]. Besides coating prosthesis with molecules, it is also possible to use a biodegradable and long-acting material which delivers molecules such as antibiotics and showed efficacy in vivo [115]. Using degradable materials may represent a good strategy, as this technique has successfully eradicated bacteria during in vivo tests [4]. For further information about innovative strategies to manage PJIs, we invite the reader to refer to Table 1.

## 4. Need for Adapted Models

As mentioned in Table 1, the potential antibiofilm strategies are very diverse. However, the method to investigate the efficiency of molecules, phages, or functionalized biomaterial is crucial, and many parameters have to be taken into account. Knowing the differences between in vitro and in vivo models and the clinical reality is determinant to examine the effect of an antibiofilm strategy. In vitro and in vivo models have intrinsic limitations to reliably mimic a real human infectious site, which consists of a complex microenvironment. In particular, the PJI infectious site is composed of various parameters such as the cells, fluids, vascularization, implants, medical conditions of the patient (e.g., possible treatments and risk factors) and, of course, the bacteria involved. All these parameters induce a complex web of possible interactions, hard to resume in simple lab models.

In vivo models are difficult to handle, time-consuming, and expensive, so in vitro models are preferentially used [34]. Nevertheless, in vitro models are sometimes too simple, and laboratory media used are often not relevant enough to the clinical reality [34]. Worse, laboratory media composition could alter behavior of antimicrobial molecules such as AMPs regarding their antibiofilm and immunomodulatory properties. For example, bone is the reservoir of 50% of the magnesium of the body, and this element is found in high concentrations in the bone environment after surgery. Indeed, the invasive gesture damages the bone and, together with the inflammatory context, promotes bone resorption and magnesium release [119]. The problem is that magnesium can interfere with AMP [88]. Thus, this parameter is essential to integrate in a screening model to assess anti-PJI strategies and highlights the priority to work with relevant models. Moreover, biofilm formation and maturation are different between the in vitro and in vivo context [120]. Indeed, biofilms are structured according to different factors, such as material and biological samples, but their structure are also highly strain-dependent, and clonal lineages can present different behaviors [95]. For patients with PJIs, the host response and localization of the bone and joint prosthesis (knee, hip, shoulder, elbow…) are also factors influencing the biofilm formation. Numerous factors are present in the bone context, and a preliminary study has shown that many of them could influence *S. aureus* biofilm formation, such as starvation, the lack of oxygen, or the presence of magnesium [121]. To investigate antibiofilm properties of a molecule or a strategy, all of these parameters should be taken into account to set up or choose the most appropriate and reliable model which satisfyingly recreate conditions from the infectious site, in order to establish more and more “personalized” research and medicine. The bone microenvironment and its dynamic make bone infection a complex subject of study that we must clearly understand to unravel new antibiofilm molecules.

## 5. Conclusions

Biofilms simultaneously harbor cells in multiple states and this physiological heterogeneity, with the matrix synthesis, are important factors in biofilm tolerance toward antibiotics and the immune system [61]. To counteract the antibiotic resistance and tolerance of *S. aureus* in PJIs by news-adapted antibiotics or other strategies, we urgently need new adapted relevant models to the host environment and the bone site. For this purpose, we have to better understand the complex relationships between bacteria and the bone microenvironment, along with the reason for bacterial attraction by prosthesis, and which factors in the bone microenvironment induce the biofilm initiation program. New strategies can involve free or coated antibiofilm molecules, immune modulation, phage therapy, and implant management. To date, most studies discussing antibiotic resistance and tolerance focus on *Pseudomonas aeruginosa*, and there is a lack of information on how much this bacterium shares these mechanisms with other bacteria, such as *S. aureus* [9,122]. However, scientists are advancing in this fight against biofilm and its tolerance to antibiotics, and several options are investigated to reduce PJI cases.

## Figures and Tables

**Table 1:** Summary of different strategies to prevent and/or treat biofilm infections discussed in recent reviews.

Strategies	Details	References
Antibiotic	Novel antibiotics and combination therapy	[31,57,101]
Other molecules	Antimicrobial peptides	[101]
	Enzymes	[31,57,116]
Immunotherapy	Monoclonal antibodies	[31,57]
Viruses	Bacteriophage therapy	[31,57,101,117]
Implant management	Coatings and surface modifications	[5,110,112,118]
Nanoparticle	Loaded or with passive passive effects	[31,57,101,110,112]
Electrical and electromagnetic methods	-	[101,112]
Ultrasound	-	[31,112]
Photodynamic therapy	-	[31,101,112]
Plasma	Non-thermal plasma	[101]
Targets	Dormant state bacteria	[31]
	Disruption of biofilm	[112]
Other	Hydrogels	[110]
	Cyclodextrin-based drug delivery	
	Titanium nanotube arrays	
